# Supporting international medical graduates’ transition to their host‐country: realist synthesis

**DOI:** 10.1111/medu.13071

**Published:** 2016-09-15

**Authors:** Amelia Kehoe, John McLachlan, Jane Metcalf, Simon Forrest, Madeline Carter, Jan Illing

**Affiliations:** ^1^ Durham UniversitySchool of MedicinePharmacy and HealthStockton‐on‐TeesUK; ^2^ University Hospital North TeesEducation and Organisational DevelopmentStockton‐on‐TeesUK; ^3^ Newcastle UniversitySchool of Medical EducationNewcastleUK

## Abstract

**Context:**

Many health services and systems rely on the contribution of international medical graduates (IMGs) to the workforce. However, concern has grown around their regulation and professional practice. There is a need, in the absence of strong evidence and a robust theoretical base, for a deeper understanding of the efficacy of interventions used to support IMGs’ transition to their host countries. This study seeks to explore and synthesise evidence relating to interventions developed for IMGs. It aims to provide educators and policy makers with an understanding of how interventions should be developed to support IMGs in their transition to the workplace, particularly looking to identify how and why they are effective.

**Methods:**

The realist synthesis involved an initial systematic search of the literature for the period January 1990 to April 2015. Secondary searches were conducted throughout the review in order to inform and test the developing programme theory. The context, mechanism and outcome data were extracted from all sources meeting the inclusion criteria. Fourteen case studies were included to further aid theory refinement.

**Results:**

Sixty‐two articles were identified, describing diverse interventions of varying intensity. A further 26 articles were identified through a secondary search. The findings illustrate that, alongside a developed programme, ongoing support and cultural awareness at organisational and training levels are crucial. Individual differences must also be taken into consideration. This will ensure that IMGs engage in transformative learning, increase their levels of self‐efficacy and cultural health capital, and reduce feelings of stress and anxiety. These factors will have an impact on work, interactions and cultural adjustment.

**Conclusions:**

Organisational, training and individual contexts all play a role in IMGs’ adjustment during the transition process. Establishing ongoing support is critical. A list of recommendations for implementation is given.

## Abstract 2

Discuss ideas arising from the article at www.mededuc.com discuss

## Introduction

International medical graduates (IMGs), those who have gained their primary medical qualifications in countries outside of the host country in which they work, have been playing an increasingly crucial role over recent years in ensuring effective delivery of health care.^1^ For example, in the UK, more than a third of registered doctors have gained their medical qualification outside the UK.^2^ This number has risen since 2014, with a particular increase in European medical graduates applying for registration.^1^

Concern has grown around the regulation and professional practice of those who qualified outside the host country. Research suggests that IMGs are likely to face difficulties with communication, culture, practical issues, team working and hierarchical structures,^3^ which in turn may lead to a greater risk of referral for fitness to practice.^4^

Although research has led to recommendations concerning the transition of IMGs,^5^, ^6^, ^7^, ^8^ the area is underdeveloped. Existing interventions, such as induction programmes and communication skills courses, are currently being implemented,^8^ often at a regional rather than organisational level. For example, half‐day programmes are often implemented without sufficient consideration of what is likely to work or how much training is appropriate. Combined with the probability that educators do not have specific experience with the particular needs of IMGs,^9^, ^10^, ^11^ this can pose a problem for those involved in developing interventions to support the transition of IMGs.

Research is needed to provide guidance on the most effective ways to implement IMGs’ transition to their new work environments, both theoretically and empirically.^8^, ^12^ Another issue to recognise is that doctors in different stages of transition will face different problems and different training needs.^13^ For example, the initial move to a host country can cause culture shock and difficulties in cross‐cultural adjustment. Once an individual has been living in the country for some time, however, they are likely to have developed personal resources, social supports and adequate coping strategies. Employers should understand the needs of their IMGs in order to support them.^14^ The induction of IMGs needs to be an ongoing, iterative process of learning that continues throughout training, perhaps through ‘buddying’ or peer support.^5^, ^14^

If resources are to be invested in ongoing, rigorous interventions, it is important to understand what interventions work within a given context and what produces the desired outcome. A recent systematic review on this subject recommended an exploration of theory and evidence in a search of educational interventions for IMGs.^8^ These processes of effective transition at the workplace level have, up until now, been taken for granted.^1^

This synthesis describes these interventions using a realist approach.^15^ It enables interventions to be fully explored, through theoretical considerations and synthesis of evidence, so that details of interventions are explored in depth to enhance understanding about why interventions work, or not, and what is likely to be effective if implemented elsewhere.

Although the focus of this synthesis is on medical graduates, graduates from other health care professions have not been excluded from the analysis. Lessons learned from looking at common forms of intervention across the health care professions can help us to further understand the conditions in which the interventions trigger differing mechanisms to generate both wanted and unwanted outcomes.^16^

## Methods

The aim of this synthesis was to explore and synthesise evidence relating to interventions developed to enable IMGs to make a successful transition to the workplace. The research aims were (i) to identify what interventions have been used to support the transition of IMGs to the workplace, (ii) to identify how and why they are effective, (iii) to identify what factors are important to achieve a successful transition and (iv) to identify the theory that explains why interventions are successful and supports wider future interventions.

### Design

Realist synthesis is a ‘systematic, theory‐driven interpretative technique’^17^ that has emerged as a strategy for synthesising evidence and providing explanations (programme theories) on why interventions may, or may not, work (i.e. how and in what circumstances). It allows interrogation of evidence from primary and secondary data sources on the contextual factors that affect successful intervention and describes the mechanisms that lead to successful intervention.^18^ Mechanisms are processes operating within an intervention that illustrate the way in which available resources are used.^15^ Key components of intervention and exploration of the context–mechanism–outcome configurations (CMOCs) were incorporated into the developing programme theory.

Realist And MEta‐narrative Evidence Syntheses: Evolving Standards (RAMESES) quality standards were used to guide the method.^19^

### Initial programme theory

Realist synthesis involves the extraction of theories from a dataset to explain the influence of a certain context on a mechanism that is being used to produce an outcome. This synthesis sought to extract theories that would explain how interventions support the transition of IMGs to the workplace. Initial theory formulation was achieved through searching relevant IMG literature, policy documents, face‐to‐face discussions with stakeholders (clinical directors from various Trusts) and discussions within the research team, who were familiar with both IMG and transition literature. Kirwan and Birchall's transfer of learning model^20^ was identified as being central to the development of initial programme theories that facilitated exploration of the concepts identified in this synthesis. The model places much emphasis on the motivation to transfer and indicates many of the elements that appear to be crucial to IMG transition. The model was also identified as being clear and applicable to the realist approach, allowing for analysis at three levels: the individual (the learner), the education or training intervention and the work environment. The focus of this synthesis was therefore on three similar contextual levels (individual, training and organisational). The transitional outcomes were based on Harrison and Shaffer's Adjustment–Effort–Performance Model.^13^ They propose three elements of transition: cultural adjustment (process of acculturation and overcoming culture shock, also associated with non‐work factors), work adjustment (performance and feeling comfortable with the job) and interaction adjustment (being comfortable with interactions with host‐country nationals). These three areas are also evident in research into transition between health care contexts.^21^ Like the transfer of learning model, it too looks at individual and organisational factors that influence job performance. These initial programme theories were used to guide data extraction and ensure the identification of relevant data. The theories were not intended to restrict theory development, but to trigger concepts and test and refine developing theory. Terms were also agreed upon at this stage to guide the synthesis and data extraction.

### Data sources and search

With the help of an information scientist, an initial systematic search was conducted to identify relevant evidence. The databases searched included Medline, PsycINFO, EMBASE and Educational Resource Information Centre (ERIC). The same key words were used across all databases relating to the target population (e.g. overseas doctor* OR International Medical Graduate*) and interventions (e.g. *support OR induction*) (see Appendix S1 for full list). It was decided that analysis would be enriched by the inclusion of grey literature, descriptive studies and theoretical papers. The results were filtered by title and then abstract by the first author. A random sample (10%) was selected and assessed by the research team to ensure quality assurance.^19^ Papers were then coded for rigour and relevance,^19^ and key findings and themes highlighted.

Titles and abstracts were considered against the inclusion and exclusion criteria, reflecting development of the initial programme theory (see Appendix S2), and duplicates were removed. Contact was made with authors via e‐mail to retrieve evaluations that had been referred to in the article but not published, or when further information was required.

### Data analysis and synthesis

Data were extracted from each study using a template that included study information, CMOCs, relevant theory, and an assessment of relevance and rigour. Relevance and rigour were assessed on a scale of 1–5 for each paper (see Appendix S3). Articles low on relevance and rigour (i.e. scored 2 or less) were generally excluded. However, descriptive pieces that provided rich data and added to theory development (high on relevance but low on rigour) were included.^19^ On occasions outcomes were implied from the mechanisms highlighted rather than explicitly stated. Extracted CMOCs were inputted into an Excel spread sheet for further analysis. Data synthesis included further refinement of the CMOCs and the generating of hypotheses. Results were extensively shared and reviewed with key stakeholders (clinical directors and policy makers), programme planning teams from health care organisations, IMGs, educators, UK doctors, educational directors and tutors. Discussions took place concerning issues such as competing theories. Realist experts from RAMESES were contacted at appropriate times to provide guidance on the realist methodology, developing CMOCs and developing theory.

Interventions were identified at the synthesis stage, from grey literature and hand searching (primarily within the UK), and were developed into case studies to aid theory refinement (a ‘reality check’). The case studies enabled further exploration of inconsistencies to test programme theories and ensure maximum variation. Discussions were held with IMGs and those involved in programme development and implementation.

The basic task of the synthesis process was to refine the programme theory^15^ (searches being iterative, not sequential).^19^ As new elements of theory were developed from the data, secondary searches for evidence to support and refine those elements were required. The research team discussed the CMOCs to develop the overarching theory.

## Results

**Figure 1:**
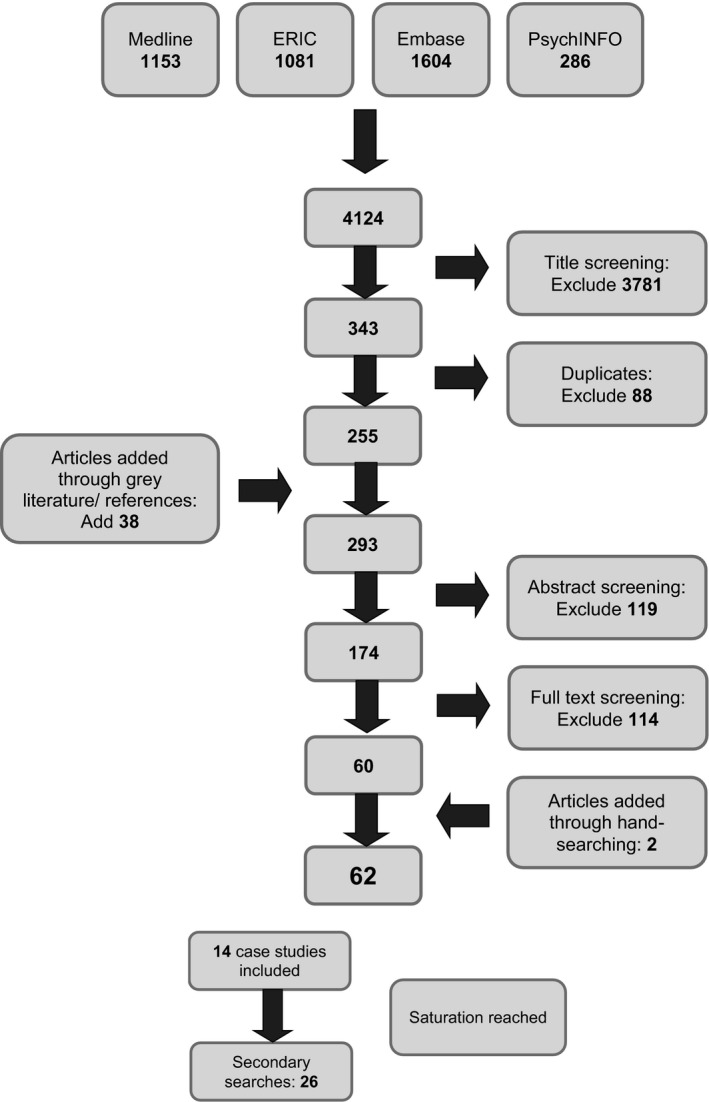
Diagram of search results

The initial search yielded 4124 results. Figure 1 illustrates the filtering process, which resulted in 62 papers being included.

The interventions included communication and linguistic skills courses, acculturation courses, examination workshops, cultural and social skills workshops, shadowing, bridging programmes, pre‐employment programmes, inductions, orientations, buddying programmes, peer support, supervised practice, ‘observerships’, simulation and web‐based resources. The articles found were from Australia, Canada, the USA and the UK. Of the 62 papers, 48 reported on doctors and 13 on nurses who graduated outside of the host country. One addressed physiotherapists. The duration of these interventions ranged from a 1‐day course (5 hours) to 3 years of training. The majority of the interventions were implemented across a number of weeks or months and included a variety of components and levels of support. All but one of the 62 papers offered ongoing support in some way, whether through support groups, buddies or ongoing training. Many regarded successful interventions to be between 3 and 5 days in length.^22^ Different kinds of transition (e.g. social and work) were generally addressed separately. The interventions focused on differing stages of transition: prior to starting work, beginning practice, and ongoing throughout employment.

Although some papers were based on rigorous evaluations of the interventions, others were merely descriptive (16 papers) and reported no outcome data. Others were small scale (*n *≤ 15) or focused on one intervention in a single setting. Both qualitative and quantitative methodologies were used, the majority consisting of self‐assessment tests before and after interventions.

As the theory developed, new areas of interest emerged and were explored further in secondary searches.^19^ Twenty‐six articles were included from secondary searches and interventions included buddying, simulation and web‐based programmes. Saturation was reached at this point as no other themes were identified to support theory development and refinement.^19^

Fourteen case studies were included in this synthesis. Each case study (CS) has been numbered and details of their duration are presented in Table 1. Interventions developed in the UK have generally been delivered over 1 day (or less). One exception was CS1, which was developed as a 5‐day programme. The three case studies from outside the UK offered ongoing support. Those from the UK largely lacked ongoing support. The case studies relied on participant feedback, but failed to evaluate the interventions independently. All but one of the interventions were aimed at doctors.

**Table 1:** Case study characteristics

Case study	Organisation	Year	Duration	Intervention
1	University Hospital of North Tees	2013	5 days	Enhanced Shadowing Programme (POD) (Pilot 1)
2	University Hospital of North Tees	2014	2 days	Programme for overseas Doctors (POD) (Pilot 2)
3	University Hospital of North Tees	2014	2 days	Programme for overseas Doctors (POD) (Pilot 3)
4	College of Physicians and Surgeons of Nova Scotia	2013	5 days	Clinician Assessment For Practice Program (CAPP)
5	Health Education North East	2010–2013	1 day	Support for overseas doctors new to clinical practice in the UK
6	Health Education North East	2014	1 day (one day follow)	Support for overseas doctors new to clinical practice in the UK
7	Registered Nurses Professional Development Centre	2012	8 sessions, 1 per week (3 hours – 6–9 pm)	Orientation to the Canadian Health Care System
8	London Deanery	2014	Online package	E‐learning Induction Package for International Medical Graduates and EU doctors
9	Central Manchester University Hospital	2014	Onine/ongoing	Online induction and support system (peer ‘buddy’ support and educational supervisors)
10	GMC	2013	1 day	Welcome to UK Practice
11	Aukland District Health Board (ADHB)	2011	26 weeks	Ready‐for‐Work Training Programme
12	Yorkshire and the Humber Foundation School	2014	1 day	Induction to NHS
13	BMA	2015	Half day	Welcome to UK Practice seminar
14	Health Education North East	2015	1 day	Communication skills for Doctors new to UK Practice

### Refined programme theory

**Figure 2:**
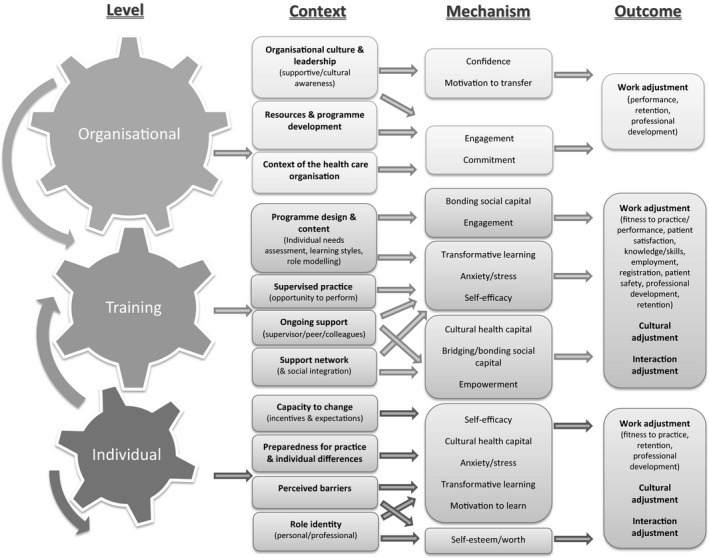
Refined programme theory illustrating IMG transition

The presentations of findings from this synthesis are framed around the three contextual levels derived from the refined programme theory (Fig. 2). The three levels (organisational, training and individual) will be described in detail, highlighting how the differing contextual factors (c) may facilitate or hinder transition, by referring to the relevant mechanisms (m) and outcomes (o).

Most of the primary outcomes found in this synthesis are related to work adjustment, such as performance,^23^, ^24^, ^25^, ^26^, ^27^, ^28^, ^29^, ^30^, ^31^, ^32^, ^33^, ^34^, ^35^ patient safety,^36^ quality of care and patient satisfaction,^25^, ^26^ retention,^25^, ^37^, ^38^, ^39^, ^40^, ^41^ staff satisfaction,^42^, ^43^ passing of assessments and registration,^44^, ^45^, ^46^, ^47^ and employment.^16^, ^34^, ^42^, ^47^, ^48^ Outcomes in terms of adjustment to life in a new country are not generally focused on, although cultural awareness was reported.^49^ Adjustment within interactions may occur through specific communication skills training and interaction with colleagues.^23^, ^30^ Cultural adjustment was largely addressed through training and the development of support networks.^28^, ^49^

### Organisational contextual factors

#### Organisational culture

The need for intercultural awareness within an organisation is essential (c),^5^, ^9^, ^16^, ^23^, ^26^, ^34^, ^45^, ^50^, ^51^, ^52^, ^53^ as IMGs arrive with differing cultural experiences and expectations that can have an impact on both learning and practice.^46^ Organisations need to understand how IMGs are coping and accept that this group may have vulnerabilities, and handle this sensitively without being patronising or creating a sense of stigma (c) (CS1). IMGs may only engage with (m) and be motivated to transfer what they have learned (m) in the programme if there is a sense of acceptance from colleagues^37^ (CS1). This will have an impact on both professional development and performance (o).

Organisations must take responsibility and be responsive to the needs of both IMGs and their institution^5^, ^12^, ^16^, ^32^, ^34^, ^37^, ^41^, ^42^, ^44^, ^47^, ^48^, ^50^, ^54^, ^55^, ^56^, ^57^, ^58^, ^59^, ^60^, ^61^, ^62^, ^63^, ^64^, ^65^, ^66^, ^67^, ^68^, ^69^, ^70^, ^71^, ^72^, ^73^, ^74^ (CS1‐10, 13), also understanding how IMGs have previously practised (c).^72^ Organisations need to hold realistic expectations and not make assumptions,^43^ either that IMGs can simply move into practice without extra support^75^, ^76^, ^77^ or cannot practise competently upon arrival^33^ (CS1‐3). Both supervisors and colleagues may benefit from cultural awareness training (c)^34^, ^64^, ^78^, ^79^ (CS6). IMGs need to feel confident (m) that the organisation in which they are working provides a high enough level of support, and a supportive culture leading to retention and high overall performance (o).^24^, ^30^, ^47^, ^52^, ^57^

IMGs should have the opportunity to build on their competencies, and not feel they must ‘unlearn’ what was previously known. A focus purely on host‐country culture can be detrimental to IMGs’ transition (c).^46^ A lack of confidence and anxiety around stigma (m) is likely to occur if employers treat IMGs as second‐rate employees.^80^ Through organisational induction, support groups, reflection and ongoing evaluation of IMGs’ adjustment,^22^ a healthier and culturally rich learning environment can be created^80^ in order for work adjustment to occur (o).

#### Leadership

A key individual, who thoroughly understands the needs of IMGs, will push interventions forward and will strive for organisational change, is needed (c)^12^, ^16^, ^23^, ^24^, ^25^, ^32^, ^47^, ^52^, ^72^, ^74^, ^81^, ^82^ (CS1‐3, 6, 9, 11, 12). It is acknowledged that there is a lack of time, resources and commitment in many cases, but an enthusiast can increase overall organisational support (CS2, 3, 6). Organisational commitment can have an impact on how supported IMGs feel, in turn increasing their levels of organisational commitment and their motivation to transfer their learning (m) (CS3) and therefore improve their performance (o).

#### Resources

The amount of resources invested into IMGs’ transition will inevitably have an impact on the characteristics of the interventions offered^9^, ^23^, ^25^, ^26^, ^29^, ^37^, ^39^, ^42^, ^44^, ^47^, ^51^, ^52^, ^55^, ^58^, ^64^, ^67^, ^69^, ^74^, ^75^, ^78^, ^83^, ^84^, ^85^, ^86^, ^87^, ^88^, ^89^ (CS1‐4, 7–9, 11). As a result of the high cost, lack of time, practicalities and inflexibility,^24^, ^84^ ongoing support may be problematic and interventions are therefore guided by available resources (c).^54^ Many failed attempts are the result of a lack of resources and time,^34^, ^46^ meaning that interventions are often implemented half‐heartedly.

Thorough and comprehensive interventions are needed to aid transition.^72^ Although a 1‐day induction is better than nothing (CS5, 6, 10), it can be viewed as more of a ‘welcome’, not offering the ongoing support needed (c)^30^, ^56^, ^63^, ^88^, ^90^ (CS12). Although resource intensive,^32^ stakeholders need to balance improved efficacy of IMGs against the amount of work carried out.^34^, ^57^ IMGs report a preference for a longer orientation,^41^ ensuring a higher level of both engagement in the acculturation process (m) (CS3) and overall benefits to adjustment (o).^54^, ^88^ Organisations should aim to draw upon local faculty members,^9^, ^12^, ^33^, ^58^ embedding programmes into their normal practice.

Engagement with the intervention may decrease if IMGs lack the income to support attendance.^48^, ^59^ Many need income to support the living costs of both themselves and their families^5^; therefore charging a fee to attend (c) is likely to reduce attendance (m)^42^, ^47^, ^79^ (CS5, 6, 14). Financial and practical support, such as accommodation and transport,^22^, ^42^ has been offered in some cases. The aim of this is to reduce the stress and financial hardship experienced by IMGs (m), although it increases the employer's costs and commitment to them.

#### Context of the health care organisation

The organisational context can affect the learning of IMGs^16^, ^22^, ^24^, ^41^, ^47^, ^67^, ^68^, ^69^, ^71^, ^72^, ^91^ (CS9); examples including training site (e.g. rural), type of speciality and level of training (c).^78^, ^82^, ^91^, ^92^ Transition can be difficult if the area in which an IMG is working does not offer the necessary resources. This is likely to result in a reduction of both commitment and engagement (m), leaving IMGs more ‘at risk’ (o).^69^ For example, rural areas may not be ethnically diverse and may not meet cultural needs, leading to feelings of isolation (m),^52^ which may lead the individual to return home (o). Conversely, smaller communities may in fact provide a good transitional environment if there is a supportive atmosphere and a sense of teamwork (c).^67^

IMGs must identify with the local community (c)^5^, ^52^ and recognise the different types of patients’ needs, social norms and colloquial language that may be specific to that area (m).^33^, ^65^ Such issues must be taken into consideration, ensuring that IMGs engage in the necessary training (m), for the well‐being of both the IMG and the patient (o).

#### Programme development

Organisations often fail to implement successful interventions because of poor development (c) (CS12), thus reducing the engagement of IMGs (m), who may feel their needs have not been addressed, and leading organisations to give up. However, thorough planning, a review of the literature and theory and responsiveness to changing needs^47^, ^70^, ^75^ will undoubtedly improve the chance of success. Programmes should not be implemented as a ‘tick box’ or for convenience.^52^ A steering group (including IMGs and experts), a pilot programme and ongoing evaluation and development are necessary to meet the needs of IMGs (o)^9^, ^12^, ^16^, ^23^, ^24^, ^33^, ^34^, ^35^, ^37^, ^41^, ^42^, ^43^, ^44^, ^46^, ^47^, ^48^, ^49^, ^53^, ^54^, ^55^, ^56^, ^57^, ^59^, ^62^, ^64^, ^65^, ^67^, ^71^, ^72^, ^73^, ^74^, ^77^, ^79^, ^82^, ^86^, ^87^, ^89^, ^90^, ^93^ (CS1‐8, 10, 12‐13).

Evidence suggests that there needs to be a better human resources (HR) process for identifying new IMGs within each organisation^12^, ^24^, ^34^, ^36^, ^41^, ^42^, ^45^, ^55^, ^57^, ^58^, ^62^, ^64^, ^71^, ^72^, ^77^, ^94^ (CS3, 4, 6‐8, 14), to ensure appropriate support and induction is put in place as early as possible and for all who require it (c).^33^ The dissemination of information in advance is crucial to the engagement of IMGs and raising awareness of potential difficulties they may face (m). Information packs sent prior to arrival in the host country may also aid adjustment to work (o),^68^, ^93^ through reducing anxiety (m), adding to perceived support and increasing initial commitment to the organisation (o).^71^ These information packs can target IMGs at the earliest phase of their transition (before arrival) and capture those who start outside of normal contractual dates.^93^

### Contextual factors of training: programme implementation

#### Individual needs assessment

The findings highlight the importance of individual needs assessment, particularly when conducted upon arrival, before any intervention is implemented (c)^2^, ^5^, ^23^, ^24^, ^25^, ^26^, ^28^, ^29^, ^33^, ^34^, ^35^, ^36^, ^37^, ^42^, ^44^, ^45^, ^46^, ^47^, ^48^, ^51^, ^52^, ^53^, ^54^, ^55^, ^57^, ^58^, ^59^, ^60^, ^62^, ^65^, ^67^, ^68^, ^70^, ^71^, ^72^, ^82^, ^83^, ^84^, ^87^, ^89^, ^90^, ^93^, ^94^, ^95^, ^96^ (CS2‐4, 7, 9). This enables training to be tailored^43^, ^56^, ^69^ and support given where necessary (CS9). IMGs will only benefit from training that is relevant and meaningful (c).^9^, ^22^ Increased *transformative learning* (self‐awareness, beliefs, behavioural) and engagement is likely to result from an initial needs assessment (m).^33^, ^56^ Organisations need to take account of the individual past experiences of IMGs within different health care systems and social and cultural contexts (c).^55^ Being aware of personal learning needs enables IMGs to reflect on these in practice (m), aiding their professional development (CS9) and resulting behaviours (o).^95^

#### Environment and learning styles

A safe environment, which is low risk and controlled, must be created when implementing interventions for IMGs (c)^2^, ^12^, ^25^, ^26^, ^32^, ^36^, ^37^, ^42^, ^49^, ^55^, ^58^, ^60^, ^67^, ^68^, ^74^, ^76^, ^81^, ^83^, ^84^, ^85^, ^93^, ^94^, ^97^, ^98^, ^99^(CS1‐7, 10‐11, 13). IMGs must feel able to recognise and admit mistakes and share challenges without fear, reducing anxiety both during the intervention and in practice (m).^29^, ^49^ In sharing experiences and reflecting on their own practice (c), IMGs’ engagement and levels of *cultural health capital* (wellbeing, resources, resilience, optimism)^100^ are likely to increase (m)^49^, and they will acquire resources to use in practice (o). Self‐efficacy is also likely to increase (m),^24^, ^87^, ^91^ so that IMGs will not blame themselves and be able to acknowledge that others are facing similar difficulties. These experiences are also likely to lead to transformative learning (m),^49^ which will further be enhanced through experiential learning involving feedback and reflection.^27^, ^46^, ^49^, ^56^ Such learning will lead to better work adjustment (o).

Experiential learning with feedback and reflection^16^, ^24^, ^29^, ^32^, ^33^, ^36^, ^37^, ^39^, ^42^, ^44^, ^45^, ^47^, ^48^, ^53^, ^54^, ^55^, ^58^, ^62^, ^63^, ^64^, ^66^, ^67^, ^68^, ^69^, ^71^, ^72^, ^76^, ^79^, ^83^, ^86^, ^88^, ^89^, ^91^, ^93^, ^97^, ^99^, ^101^ (CS1‐3, 7, 11, 13), such as simulation^70^, ^77^ and role play,^102^ creates active participation (c), enabling IMGs to learn through errors, problem solving and skill rehearsal^61^, ^87^ and relate these to real life practice (m).^34^ Professional self‐efficacy will increase (m), enabling IMGs to feel more able to apply new knowledge and skills in practice (o)^29^, ^87^ (CS1‐3). They will acquire new resources that can be used in what are potentially stressful situations (m), causing them to feel more prepared to meet the demands of practice (o).^29^, ^77^, ^83^ Educators must be aware of differences in learning styles and learner responsiveness (c).^27^, ^56^, ^70^

Learning is both an individual and social experience and small group discussion and peer feedback are essential (c)^12^, ^16^, ^23^, ^24^, ^26^, ^32^, ^33^, ^37^, ^46^, ^48^, ^49^, ^55^, ^56^, ^57^, ^58^, ^62^, ^65^, ^72^, ^78^, ^79^, ^83^, ^87^, ^88^, ^89^, ^91^, ^94^, ^96^, ^97^, ^99^, ^102^ (CS1‐3, 6, 10, 14), as they promote self‐reflection and self‐awareness (transformative learning) (m). Engagement is much more likely to occur in small groups than in larger groups (m)^77^ once initial shyness is overcome.^62^, ^87^ Identity formation within the group is likely to occur (m) through sharing of stories and experiences^62^, aiding both work and cultural adjustment (o).

#### Role modelling

The inclusion of role modelling is seen as important for IMGs^2^, ^12^, ^24^, ^33^, ^36^, ^38^, ^40^, ^42^, ^49^, ^50^, ^53^, ^70^, ^71^, ^73^, ^75^, ^78^, ^81^, ^83^, ^84^, ^85^, ^87^ (CS4, 6, 7, 9, 10), as it enables them to relate to those who have faced similar issues (c).^22^, ^60^ Hearing the stories of IMGs who have been through a similar transition will raise awareness that issues faced are ‘universal’, reducing anxiety and increasing cultural health capital (m).^37^ Seeing others succeed may also increase self‐efficacy (m)^22^, ^87^ and lead to work adjustment (o).

#### Programme content and design

The programme content must be relevant to IMGs and reflect issues of concern to them^5^, ^23^, ^24^, ^27^, ^32^, ^33^, ^35^, ^37^, ^40^, ^41^, ^42^, ^44^, ^46^, ^47^, ^48^, ^49^, ^51^, ^54^, ^56^, ^57^, ^61^, ^62^, ^65^, ^67^, ^69^, ^70^, ^71^, ^72^, ^73^, ^75^, ^76^, ^82^, ^87^, ^89^, ^93^, ^97^, ^103^ (CS1‐7, 9, 13, 14), being learner focused and meeting individual needs (c)^32^, ^56^ (CS2, 3, 10). IMGs are more likely to engage with the content (m) if a rationale for learning is identified. This will then lead to increased knowledge, skills and understanding^16^, ^23^, ^27^, ^30^, ^33^, ^35^, ^44^, ^45^, ^46^, ^48^, ^49^, ^56^, ^57^, ^59^, ^66^, ^67^, ^70^, ^73^, ^79^, ^89^ in areas such as communication and language, professionalism, cultural awareness and development of clinical and organisational workplace competence (o).

Interventions should not be built purely on deficiencies in medical knowledge and communication^22^, ^60^, ^70^ (CS11), but should include the professional role^90^ and survival skills to enable future difficulties to be managed succesfully.^9^, ^60^, ^79^ Organisations often focus on clinical competence and ignore crucial learning needs.

### Contextual factors of training: external to programme implementation

#### Ongoing support (training, peer and supervisory)

Ongoing support is central to IMGs’ transition (c)^2^, ^12^, ^16^, ^25^, ^26^, ^28^, ^30^, ^32^, ^34^, ^35^, ^36^, ^37^, ^38^, ^40^, ^41^, ^44^, ^47^, ^50^, ^53^, ^55^, ^56^, ^59^, ^62^, ^64^, ^67^, ^68^, ^70^, ^71^, ^72^, ^74^, ^75^, ^76^, ^77^, ^78^, ^81^, ^83^, ^84^, ^85^, ^86^, ^87^, ^90^, ^91^, ^101^ (CS7, 9, 11), helping them to manage stress (m) and adjust in a healthy manner.^104^ High levels of support at an early stage^26^, ^40^ would be most beneficial as they will help identify any initial problems and prevent escalation (c).^22^ Monitoring of progress after an induction programme^22^, ^58^ will ensure transfer of learning (m) and fitness to practice (o)^16^, ^87^ (see Appendix S4).

Implementing a buddying or mentor scheme is an effective way to provide ongoing support, enhancing the efficiency of an initial programme^42^, ^52^, ^67^, ^69^, ^85^, ^87^, ^104^ without being costly. The needs of the individual will determine whether they will benefit from a host‐country buddy^52^ (CS2, 3) or an IMG buddy^53^, ^90^ (CS9). Both have advantages and disadvantages, offering different types of support. Having a personal buddy who can provide information when necessary (c) will reduce stress and anxiety and support cultural health capital (m).^37^, ^38^, ^103^ An increase in self‐efficacy (m) is likely to result from this support^26^, ^37^, ^87^ (CS2), this confidence ultimately being applied in the learning environment (o).^67^ Retention is also more likely, with the ability to adapt to and master new skills (o).^47^, ^87^

Supervisors are key to providing ongoing support and regular feedback (c).^2^, ^5^, ^9^, ^23^, ^27^, ^32^, ^34^, ^37^, ^42^, ^44^, ^47^, ^48^, ^54^, ^58^, ^59^, ^64^, ^65^, ^67^, ^73^, ^74^, ^76^, ^78^, ^83^, ^87^, ^89^, ^101^ Supervisors should be involved with both the implementation of the intervention and individual needs assessment,^47^ aiding transformative learning (m) and providing a platform for continued professional development (o)^58^, ^67^, ^85^ (CS9). The quality of supervision is important, as IMGs often require more support than host‐country graduates,^2^, ^32^, ^54^, ^76^ which can be time consuming^27^, ^67^ (CS1‐3).

Peer support in practice is also essential to ensure the necessary support is available.^25^, ^26^, ^33^, ^35^, ^42^, ^47^, ^48^, ^53^, ^54^, ^56^, ^67^, ^83^, ^84^, ^97^ It is noted that there is often a lack of support and cultural awareness amongst colleagues^55^, ^64^, ^78^, ^79^ (CS6, 14), which may lead to a risk of stereotyping, prejudice and discrimination (c)^34^, ^50^, ^52^, ^96^ (CS14). Where there are perceptions of hostility, this can hinder acquiring social capital and lead to stress, anxiety, feelings of exclusion and poor cultural health capital (m).^34^, ^52^, ^55^ This impact upon resilience in overcoming difficulties^55^, ^79^ will hinder both performance and retention of knowledge (o).^52^ For example, if there is no support from supervisors or peers, IMGs may feel unable to apply the skills they learned within the training, such as communicating with colleagues, so will not ask questions if they are unsure in practice (o)^52^ (see Appendix S5).

Colleagues must understand both equality and diversity and feel able to give feedback to IMGs without the fear of being seen as discriminating. Where colleagues illustrate empathy and support,^82^ being appreciative of past experiences (c), social capital is much more likely to increase, impacting intercultural communications and cultural health capital (m).^37^, ^38^, ^55^, ^103^ This support is also likely to increase feelings of acceptance, empowerment and both personal and professional self‐efficacy (m)^37^, ^55^, ^67^, ^77^, ^81^, ^87^ (CS4). Transfer of learning is more likely to take place (o).^34^, ^55^

#### Supervised practice

Opportunities to perform the learned skills in practice, with feedback (c), will both reinforce learning and aid in behaviour change (transformative learning) (m).^59^, ^76^, ^79^ All team members need to be involved in supporting IMGs if they are to transfer what has been learned in the intervention and ultimately be prepared for practice (o).

#### Support network (including social integration)

Support networks should consist of IMGs and host‐country graduates (c) to ensure that both bridging and bonding social capital occurs (m)^12^, ^97^ (CS7, 10). Silo social development hinders the ability to communicate with colleagues (o).^46^, ^55^ IMGs will benefit from learning through social interactions with all groups (c),^12^, ^25^ which will increase self‐efficacy and resolutions of personal conflicts and lead to expressions of feelings (m).^78^ However, where there are feelings of isolation and hostility from colleagues (c), there can be a dependence on those from the same culture (m) (CS6). A well‐resourced work environment is needed (c).^12^, ^26^ This helps IMGs identify and connect with their network and has an impact upon transformative learning, particularly negotiation and awareness of differences (m).^12^, ^49^, ^60^, ^67^, ^78^ This will in turn lead to greater levels of professional development (o).^12^, ^78^, ^89^ Both IMGs and host‐country graduates will benefit from bridging social capital (m), creating access to new resources^12^, ^60^ that will enable more positive behaviours within the workplace (both work and interaction adjustment) and increase retention (o).^25^, ^40^

### Individual contextual factors

#### Capacity to change

Commitment to change is necessary,^57^ IMGs having to participate in and understand the need for training (c)^26^, ^34^, ^38^, ^39^, ^42^, ^45^, ^47^, ^49^, ^56^, ^61^, ^66^, ^67^, ^72^, ^75^, ^81^, ^83^, ^85^, ^87^, ^96^, ^104^ (CS1‐4, 6, 12) if transformative learning is to take place (m). If IMGs are not accepting of the new culture (c),^52^ bridging of differences between the two cultures (m)^55^ and overall work adjustment (o) will not take place.

Holding an unrealistic self‐image will also have an impact on this capacity to change (c)^12^, ^26^, ^45^, ^46^, ^58^, ^66^, ^70^, ^76^, ^78^, ^79^, ^82^, ^86^, ^97^ (CS2, 12). This lack of self‐awareness is likely to result in reduced motivation to learn (m),^48^, ^62^, ^63^, ^71^ for example wanting to complete the programme in the shortest possible time,^34^ and resistance to feedback (m).^43^ Many IMGs focus on acquiring medical skills and knowledge and so fail to see the need for other training,^60^, ^62^ which has an impact on their professional development (o).^51^, ^85^

#### Incentives and expectations

Incentives for IMGs to participate in a programme (c) will have an impact upon motivation to learn (m) and transfer into practice (o)^12^, ^24^, ^30^, ^35^, ^36^, ^39^, ^41^, ^44^, ^48^, ^54^, ^55^, ^58^, ^60^, ^65^, ^67^, ^69^, ^72^, ^79^, ^83^, ^85^, ^87^, ^91^ (CS2‐4, 7, 11). An individual motivated by the outcome of exams (c) may focus heavily on this^32^, ^47^, ^57^, ^89^, ^102^ (CS9) and be less motivated to improve practice (m).^46^, ^67^ They may learn within the assessment context but not necessarily transfer this to practice (o). If IMGs participate when attendance is voluntary (CS3, 5, 6, 14) or there is a fee requirement (c)^47^ (CS11), their motivation to learn is likely to be high (m), whereas mandatory attendance or not requiring a fee (c) (CS1‐3, 12) may produce a less motivated group (m). However, if there is a lack of insight into the new ways of practising or if IMGs do not have the self‐awareness to assess their own needs, individuals will not participate voluntarily (c) (CS2) and transformative learning will not occur (m). Without incentives, individuals may be less motivated to learn (m) and more susceptible to drop out (o).^67^ As discussed at the organisational level, where high commitment and support is demonstrated by the organisation, the incentive to learn is also likely to increase (m).^32^, ^68^, ^93^

IMGs often make real sacrifices to take up job opportunities in another country, either leaving family behind or uprooting them (CS1‐3, 6, 14); therefore, they are likely to arrive highly motivated. However, this motivation may not last if IMGs arrive with unrealistic expectations and goals^54^ with regard to quality of life, work experiences and their future employment opportunities (c).^48^, ^89^ IMGs must arrive aware of cultural and organisational expectations^42^, ^44^, ^45^, ^46^, ^67^, ^68^, ^72^, ^96^ (CS1‐4, 6, 9, 10, 14), otherwise they are likely to face culture shocks that will hinder the acculturation process (m)^52^ and overall work adjustment (particularly performance and retention) (o). Training and support programmes can therefore aid in explaining the expectations of both the organisation and the culture, and in developing realistic career goals (c)^56^, ^89^ and highlighting any potential barriers to practice (m).^48^ IMGs may experience changes in self‐esteem, related to these unrealistic aspirations and the discrepancy with achievement (m),^80^ which will affect their professional development within the organisation (o).

#### Role identity

Unrealistic expectations can also be linked to role identity^26^, ^38^, ^45^, ^48^, ^64^, ^65^, ^67^, ^71^, ^72^, ^74^, ^76^, ^78^, ^86^, ^87^, ^101^ (CS2, 6); for example, if IMGs arrive and have to take up posts that are a lower grade than expected or a different specialty^78^. The job role in comparison to their previous role may be lower in status and power,^34^, ^42^, ^54^, ^96^ perhaps resulting in personal, cultural and professional loss of status and identity (c).^41^ Taking up the role of learner^42^, ^54^ may lead to perceptions that they are unable to use their current skills,^2^, ^34^ must deskill or ‘unlearn’ their old ways and conform to practice,^59^ reducing self‐efficacy (m).^42^ IMGs have reported feeling unappreciated, targets of racism and being seen as a ‘group’^67^ rather than individuals (c), further hindering self‐efficacy (c). IMGs have also reported that their qualifications are not recognised by their peers or employer (c).^72^, ^78^ If this is the case, motivation to learn is likely to be low and resistance high (m).^96^ IMGs must be supported to maintain a positive self‐image^67^ and seek to establish themselves in their new social and learning environments (c), which are likely to be different to what they are used to. Combining both old and new skills^34^ will reframe their professional identity and enable them to modify practice (m) and take control, leading to professional growth and cultural adjustment (o).^41^, ^96^

If IMGs feel they need to ‘prove’ themselves (c)^42^, ^58^, ^63^ (CS2), their levels of self‐esteem and self‐worth may be affected (m).^60^, ^72^ The feeling of not belonging, as well as not wanting to appear incompetent or weak by admitting difficulties (c),^58^, ^94^ may lead to increased anxiety^38^ and communication barriers (m), which will hinder interactions with colleagues (o).^59^

#### Preparedness for practice

IMGs will have varying levels of clinical knowledge and experience (including learning styles)^12^, ^16^, ^23^, ^24^, ^28^, ^29^, ^31^, ^41^, ^45^, ^48^, ^52^, ^54^, ^56^, ^58^, ^59^, ^61^, ^64^, ^65^, ^67^, ^68^, ^70^, ^71^, ^72^, ^76^, ^77^, ^78^, ^79^, ^83^, ^89^ (CS1‐4, 7, 11). A lack of clinical knowledge and competence^32^, ^46^ is likely to hinder interventions for IMGs as their focus will be on lack of knowledge (c), rather than the learning outcomes of the intervention (m)^59^ (CS2, 3). Educators will expect a certain level of competence and therefore will incorporate this into the content.^33^ Differences in previous practice must also be acknowledged (c), an initial reluctance to question seniors or share views during ward discussions perhaps hindering transformative learning (m) and overall practice (o).^34^

#### Individual differences

Individual differences are likely to play a role in transition^5^, ^12^, ^26^, ^34^, ^40^, ^42^, ^47^, ^49^, ^54^, ^62^, ^70^, ^75^, ^83^, ^84^, ^96^, ^99^, ^104^ (CS1‐4, 6, 12). The acculturative process is mediated by individual factors such as age, gender, ethnicity, culture, personality, etc..^26^, ^27^, ^37^, ^59^, ^102^, ^105^ For example, female IMGs, who often have differing needs to men, may need different support (c). ‘Home’ gender roles and gender role expectations may be in conflict with those in the new culture (m)^105^. However, women have been reported to experience fewer feelings of isolation and higher levels of engagement (m).^37^ Transition is likely to be influenced by the personality of the individual and how resilient they are (c), culture shock being experienced by some individuals but not others (m).^26^ It is essential to be aware that culture shock has been linked to poor mental health (o).^89^ Individual differences (c) may therefore have an impact on acculturation (m) and create a barrier to practice (o).^48^ Individual differences may also limit the emotional support offered by educators because of a fear of violating gender or cultural boundaries.^79^ The differences between those from international and European countries must also be recognised (CS1‐3); for example, attitudes to mistakes and feedback (m).^99^

There may well be differences in individual circumstances that have an impact on adjustment^41^, ^52^, ^59^, ^71^, ^75^, ^76^, ^89^(CS1‐4); for example, the nature of migration, barriers faced in gaining employment and length of time living in the host country (c).^101^ The process of migration itself may be stressful (m), both practical issues and lack of a social network impacting on this (c).^67^, ^80^ For example, if the individual has a spouse that has moved with him or her, or family and friends already living in the host country^101^ (CS2, 6), he or she may be able to adjust more quickly (o). Acculturation can also be hindered by family issues, such as spouses or children finding it difficult to adjust.^60^

#### Perceived barriers

IMGs may feel that the system is unfair, particularly where barriers make entering the system and career progression difficult.^67^, ^68^, ^78^, ^89^ A programme for international graduates may therefore be seen as another hurdle that they have to clear (c),^59^ perhaps decreasing motivation to learn (m). Being told that extra training is needed can have an impact on self‐esteem (m)^55^ and ultimately work adjustment (o). Barriers may also be perceived following training. For example, a sense of being unnecessarily questioned (c) will act as a threat to the sense of self (m).^96^

## Discussion

This synthesis has investigated interventions designed to aid the transition of IMGs to health care organisations, specifically looking at contextual factors that will mediate mechanisms that are likely to lead to a successful transition (Fig. 2). Successful interventions seem to be those that not only offer a developed programme, targeting individual needs of the IMGs within a supportive learning environment, but offer ongoing support from both peers and supervisors during and following implementation. These factors are key in prompting IMGs to engage in transformative learning, increase their self‐efficacy and cultural health capital, and reduce feelings of stress and anxiety. Where cultural awareness and support of the organisation are evident, interventions are likely to be more successful, with increased engagement, motivation and commitment from IMGs. Interventions will be most effective for those individuals who have the capacity to change and maintain their role identity; organisations have to understand that IMGs will be making their transition with differing levels of preparedness and individual differences. Organisations must aid IMGs with their loss of natural support networks, feelings of isolation and pressure to take on a new culture.^60^

Findings were organised into three contextual levels, individual, training and organisational, thus developing the initial programme theory. The findings were presented in an applicable manner, focusing on context as the synthesis intended to provide guidance for those implementing programmes for IMGs. This is primarily important for educators currently implementing or developing their own interventions. At a regional level, policy makers can use the findings to develop guidelines that health care organisations can use at a local level. Programmes are currently being implemented within some organisations, but if the necessary contextual factors are not embedded, the necessary mechanisms may not be triggered, so transitional outcomes (in terms of adjustment) may be hindered. It should be the responsibility of both local and regional levels to ensure these issues are addressed.

Providing a variety of safety nets, such as induction, buddying and enhanced supervision, is crucial for successful transition, supporting recommendations from earlier research.^7^ There are more issues to address than merely lack of skills. A smooth transition takes about 1 year^59^ and resources are needed once IMGs begin.^35^ Stress is likely to be high for this group of doctors facing challenges both inside and outside of work. Dysfunctional adjustment may not only lead to poor job performance, but also result in IMGs resigning,^13^ which will have an impact on organisations.

As the ‘cogs’ illustrate in Figure 2, the most efficient transition process will occur if all levels are operating effectively. Although adjustment will take place at the individual level, inputs at the organisational and training levels are crucial (see Appendix S4 and Appendix S5 for examples). The complexity of issues at all levels must be understood in order to ensure transfer of learning into practice.

Although programme content and delivery are important, an organisational culture that creates a social support network and a feeling of being welcomed and accepted stands out as having the biggest impact on IMGs’ transition.^106^ Individual organisations should be encouraged to develop and pilot interventions, taking into account the recommendations given in Table 2.

**Table 2:** Recommendations for implementing interventions for IMGs

An individual needs assessment is crucial prior to induction, taking into account individual differences and context of the health care organisation.
Information concerning work, cultural and general issues should be sent to IMGs at the earliest opportunity.
A rigorous and mandatory induction should be provided as early as possible before IMGs begin practice; content should include communication skills, cultural awareness, and practical and ethical guidelines, with follow‐up sessions throughout the year ensuring reflective practice. The induction must be flexible to accommodate the identified needs.
Those who are not in training posts should be given the same induction and level of support.
Supervisors should be made aware of individual needs and give ongoing feedback to IMGs.
Differing learning styles and cultural experiences must be taken into consideration.
Experiential learning opportunities are needed to ensure new practices are adopted and transferred into practice.
A buddy should be assigned upon arrival to provide ongoing support.
Cultural awareness within the organisation must be evident.
A robust HR process needs to be in place to ensure IMGs entering the organisation are identified.

### Strengths and limitations

The realist approach is emerging as a popular choice of methodology for understanding intervention effects in context. It is an exciting, iterative approach, which involves the negotiation between stakeholders and researchers, adding more depth than the traditional systematic approach. As a result, a vast number of context‐mechanism‐outcome patterns were drawn from the findings in order to build and refine the developed programme theory. This synthesis has begun to extend the knowledge base in the area of IMG transition, filling the gap that has previously been noted.^8^ The detailed exploration has gone beyond the findings from a regular systematic review,^8^ providing much needed theoretical explanations as to how interventions achieve their effects, and not just drawing conclusions from empirical generalisations. This synthesis does not offer particular lessons learned from best practice or a recommendation of the ‘best’ interventions for IMGs, but instead offers a tailored and transferable programme theory through a process of theory building. Knowing how interventions for IMGs work may prove more valuable than single recommendations in ensuring success. All of the interventions vary in terms of what they offer the IMG transition process; therefore in synthesising the data, the researchers were able to see what elements are crucial to successful outcomes and make recommendations for best practice.

It further highlights the vast differences in the ways interventions are being developed both within and between different countries. Therefore it is suggested that a standardised set of guidelines is developed within each country, using recommendations made from this research, to ensure effective development and implementation of interventions.

A limitation of this synthesis was the lack of objective outcome measures within the literature. The majority of papers that reported outcomes were reliant on satisfaction surveys or self‐assessments, which are known to be unreliable and problematic,^107^ or were purely descriptive. Small sample sizes were also common. This highlights a weakness in the current literature on IMG transition, also noted in a recent literature review.^8^ Therefore there was a lack of complete rigour in the conclusions drawn, the researchers being unable to test all aspects of the programme theory in detail. However, when outcomes were lacking, they were inferred. The use of secondary searches also helped to refine theory where lacking. Where outcomes were explored in a rigorous manner, studies often lacked the rich description needed to explore the theory in detail. A realist evaluation of primary data is currently being conducted to aid in further refinement and development of the proposed programme theory.

An overlap between categories, particularly mechanisms and outcomes, arose on a number of occasions, illustrating the subjective nature of the realist approach. However, these were discussed extensively and realist experts consulted when facing difficulties.

Case studies were largely limited to the UK as this was the context of the wider research. They did, however, highlight that the UK is lacking the ongoing support that other countries seem to offer IMGs. Because ongoing support is a core theme from the findings, this is something that needs addressing. Although the theory was tested in a UK context, there is international relevance, in the same way that we have benefitted from international experiences in conducting the work.

As discussed previously, it was decided that graduates from different health professions would be included, despite the focus of the synthesis being on medical graduates. Future research may therefore wish to look at the transition of other professions separately. There could, however, be an opportunity to develop interventions within organisations for all international health professionals. As this synthesis shows, the transitional needs of doctors and nurses are relatively similar and may aid in acculturation.

What still remains unclear from the findings is how to target IMGs who start outside of normal contractual dates, interventions largely being implemented yearly. The suggested ongoing support, such as buddying, online resources and information packs prior to arrival may help to overcome this issue, but further exploration is needed.

## Conclusions

Designing interventions for IMGs is a complex task;^108^ therefore, this synthesis describes the theory and processes needed to support IMGs to make a successful transition to their host country. The findings illustrate why interventions work (or not) and in what ways, thereby enabling those implementing interventions to make more informed choices. Systematic reviews rarely look at the underlying theories of interventions or explain the context, mechanisms and outcomes that will aid in future implementation.^18^ Contextual factors at three levels, organisational, training and individual, have been proposed, and the particular importance of their interactions highlighted. Ongoing support and cultural awareness at both the organisational and training levels are crucial, whilst taking into consideration individual factors. Organisations should not expect a quick or easy transition, or ignore potential difficulties at any of the proposed levels.

## Contributors

AK: led the piece of research, finalising the conception and design of the work; conducted the case studies included in the research; did the majority of the analysis work, led discussions and finalised the interpretations; made final revisions to the work and gave approval for the submitted version. JI: involved in the design, analysis of the work and revising the content. JM: involved in the analysis, interpretation of the findings and revising the content. MC: involved in the analysis and interpretation of the findings. SF: involved in interpretation of the findings and revising the content. JM: developed the initial protocol for the research and contributed to the design of the work. All authors contributed to the drafting and revision of the paper. All agreed to be accountable for all aspects of the work after final approval of the version to be published was given.

## Funding

North Tees and Hartlepool NHS Foundation Trust.

## Conflicts of interest

none.

## Ethical approval

full ethical approval was granted by Durham University School for Medicine, Pharmacy and Health Ethics Committee.

## Supporting information


**Appendix S1**. Search terms.
**Appendix S2**. Table of inclusion and exclusion criteria.
**Appendix S3**. Scale for assessing relevance and rigour of papers.
**Appendix S4**. Example of CMOc.
Use of theory to explain why Gerrish & Griffith (2004) reported successful intervention outcomes
**Appendix S5**. Example of CMOc.
Use of theory to explain why one individual from case study reported transition struggle.
