# Supplementary material for: Supporting international medical graduates’ transition to their host‐country: realist synthesis
Source: Med Educ. 2016 Sep 15;50(10):1015–32. doi: 10.1111/medu.13071 (PMC5113661; doi:10.1111/medu.13071)
Supplement: Supplementary file 1 — Appendix S1. Search terms. Appendix S2. Table of inclusion and exclusion criteria. Appendix S3. Scale for assessing relevance and rigour of papers. Appendix S4. Example of CMOc. Use of theory to explain why Gerrish & Griffith (2004) reported successful intervention outcomes Appendix S5. Example of CMOc. Use of theory to explain why one individual from case study reported transition struggle. [file MEDU-50-1015-s001.docx]

***Appendix 1 (Search terms)***

*IMG terms*

Overseas doctor* OR International Medical Graduate* OR IMG* OR non-UK graduate* OR non-UK qualified OR Overseas Medical Graduate* OR Overseas Graduate OR foreign doctor* OR immigrant doctor

*Intervention terms*

Support OR training OR induction OR program* OR intervention OR introduction OR orientation

*Specific interventions terms*

Simulation OR buddy* OR mentor* OR shadow*

***Appendix 2 (Table of inclusion and exclusion criteria)***

| **Inclusion** | **Exclusion** |
| --- | --- |
| Intervention within healthcare setting | Limited description of intervention |
| Target population is overseas graduates | Limited analysis/results (intervention not ran) |
| Relevant intervention (transition/support) | Published before 1990 |
| Full article | Not in English Language |
| Papers may be opinion based/suggestions through research | Not within healthcare setting |
| Case Studies not published in literature | Intervention aim is not to aid transition |
| Must be generalisable to all overseas graduates | Undergraduate university programme |
| Any country considered | Focus of intervention should not be competency/knowledge based only |
| Any duration (aim being transition/support throughout) | Findings cannot be generalised to overseas graduates |

***Appendix 3 (Scale for assessing relevance and rigour of papers)***

***Test relevance*** –Is the evidence provided in this theory area relevant enough to be included in the synthesis?

5 - Direct relevance to transition interventions that had been evaluated within a healthcare context and can therefore contribute to theory building and/or testing.

4 – Relevance to transition interventions (provides evidence around theory, has not necessarily been fully evaluated).

3 – Relevant concepts and theories.

2 – Potentially relevant concepts and theories.

1 – Does not address the theory under test (no relevance can be made to transition).

***Test rigour*** –

5 - Methods used to generate the relevant data are credible and trustworthy and fully support the theory.

4 - The research supports the conclusions drawn from it by the researchers or the reviewers but methodology could be improved.

3 – Provides valuable contribution to the conclusions drawn, despite some flaws in methodology.

2 – Provides some contribution to conclusions drawn despite poor methodology.

1 - Methods used to generate the relevant data are not credible and untrustworthy and provide no contribution to any conclusions drawn.

***Appendix 4 (Example of CMOc)***

***Use of theory to explain why Gerrish & Griffith (2004) reported successful intervention outcomes***

The organisational culture is one that values diversity and equality of opportunities (c). Despite being resource intensive, the need for the adaption programme is recognised (c). The programme was well developed and offered ongoing support through supervised practice and a mentor (c). Despite a perceived lack of supervisor time (c); causing them to not ‘learn properly’, and feeling ‘unappreciated’ and different, the overall ongoing support overcame any potential issues here. Individual incentives for participation were initially focussed on exam success, a loss of status motivating them to gain registration as soon as possible (c). However through the developed programme and ongoing support, the overseas graduates recognised that they have other needs that have to be addressed in order to be fit for practice (m). This support also led to greater feelings of ‘self-efficacy’ as they perceived themselves to be a valued part of the team (m). As a result, successful outcomes included registration, fitness for practice and increased retention rates (o).

This example illustrates the interaction of the three contextual levels. The organisational culture and level of support, not only leads to increased confidence and commitment from the overseas graduate themselves (m), but feeds into the development of the programme and ongoing support offered at the training level (c). Where contextual issues arise that may hinder adjustment, such as individual incentives, perceived underlying racism or strain on resources, the ongoing support at the training level enables the necessary mechanisms to be triggered (self-efficacy and transformative learning) which in turn may lead to registration, fitness for practice and retention (o).

***Appendix 5 (Example of CMOc)***

***Use of theory to explain why one individual from case study reported transition struggle***

The individual is motivated to learn and the organisation is supportive in terms of training (c). However, the individual states that peers and supervisors are unsupportive (c). This has led to a lack of self-efficacy and feelings of stress (m). The individual will not ask questions in practice, further reducing cultural health capital (m) and hindering professional growth (o). Not asking questions has also led to patient safety issues, the individual performing procedures without knowing how to do so (o). Perceived barriers and feeling of loss through poor treatment (c) may lead to feelings of low self-esteem and self-worth (m) and may explain why other IMGs have returned home (as stated by the individual) (o).

Therefore, a lack of support (c) may lead to reduced self-efficacy, feeling of loss, and increase in stress as they feel unable to ask questions or find necessary resources (m), impacting on their performance in the workplace and increase the chances they will return home (o).
